# Diversity and Composition of the Skin, Blood and Gut Microbiome in Rosacea—A Systematic Review of the Literature

**DOI:** 10.3390/microorganisms8111756

**Published:** 2020-11-08

**Authors:** Klaudia Tutka, Magdalena Żychowska, Adam Reich

**Affiliations:** Department of Dermatology, Institute of Medical Sciences, Medical College of Rzeszow University, 35-055 Rzeszow, Poland; klaudia123191@vp.pl (K.T.); magda.zychowska@gmail.com (M.Ż.)

**Keywords:** microbiome, microbiota, rosacea, skin, blood, gut

## Abstract

Rosacea is a chronic inflammatory skin disorder of a not fully understood pathophysiology. Microbial factors, although not precisely characterized, are speculated to contribute to the development of the condition. The aim of the current review was to summarize the rosacea-associated alterations in the skin, blood, and gut microbiome, investigated using culture-independent, metagenomic techniques. A systematic review of the PubMed, Web of Science, and Scopus databases was performed, according to PRISMA (preferred reporting items for systematic review and meta-analyses) guidelines. Nine out of 185 papers were eligible for analysis. Skin microbiome was investigated in six studies, and in a total number of 115 rosacea patients. Blood microbiome was the subject of one piece of research, conducted in 10 patients with rosacea, and gut microbiome was studied in two papers, and in a total of 23 rosacea subjects. Although all of the studies showed significant alterations in the composition of the skin, blood, or gut microbiome in rosacea, the results were highly inconsistent, or even, in some cases, contradictory. Major limitations included the low number of participants, and different study populations (mainly Asians). Further studies are needed in order to reliably analyze the composition of microbiota in rosacea, and the potential application of microbiome modifications for the treatment of this dermatosis.

## 1. Introduction

Rosacea is a chronic inflammatory skin disease that commonly affects white, middle-aged females. The prevalence varies with population, and the disease is infrequently reported in nonwhite patients. According to recent studies, the incidence of rosacea was estimated to be 25.6% in the older Finnish population [1], while in China the incidence rate was found to be 3.4% [2]. Rosacea is characterized by the presence of periodically intensifying centrofacial erythema, often associated with teleangiectases (erythematoteleangiectatic rosacea, ETR) or inflammatory papules and pustules (papulopustular rosacea, PPR) [3]. The pathophysiology is not fully understood, and several factors are believed to contribute to the development of the disease, including neurovascular reactivity, genetic susceptibility, dysfunction of the innate immune responses, and comorbid gastrointestinal conditions [3,4,5].

As antibiotics and ivermectin are successfully used to treat rosacea, one can speculate, that microbes may play an important role in the pathophysiology of the disease. Several microorganisms (e.g., *Demodex* spp., *Cutibacterium acnes*, and *Staphylococcus epidermidis*) have been identified in rosacea subjects, using classical isolation methods [6]. Still, the exact disturbances that lead to the development of the condition remain unknown, which prevents the use of targeted therapy.

Microbiome is defined as the total pool of microorganisms, their genomes, and interactions in a given niche [7]. In recent years, tremendous progress has been made in the utilization of metagenomic methods based on the analysis of 16S ribosomal RNA (rRNA) for investigating the human microbiome of the skin and gastrointestinal tract, and its association with chronic dermatoses. A novel approach, and superior to the culture-based studies, it enables more thorough analysis of the intra- and inter-sample diversity of microbiota, referred to as α- and β-diversity, respectively. Skin and gut microbiota were initially the subjects of the majority of studies, as the role of the gut–skin axis is widely-recognized, but not fully understood. Metagenomics has already been utilized for the investigation of the changes of skin and gut microbiome in atopic dermatitis [8], acne [9], and psoriasis [10]. A relatively novel approach is also the metagenomic analysis of the alterations of blood microbiota, which have so far been overlooked by the culture-dependent methods. This concept has recently been proposed for explaining the link between the gut and skin microbiomes in several dermatological conditions, including hidradenitis suppurativa [11].

The objective of the current paper is to summarize, and critically review, the so far reported alterations in the microbiome of the skin, peripheral blood, and gastrointestinal tract in patients with rosacea.

## 2. Materials and Methods

### 2.1. Search Strategy

A systematic review of three medical databases (PubMed, Scopus, and Web of Science) was performed in accordance with the PRISMA (preferred reporting items for systematic review and meta-analyses) guidelines. The three databases were searched in August 2020 for studies that aimed to investigate the role of the microbiome (skin, gut, and/or blood) in rosacea. The following search criteria were used: “rosacea” combined with “microbiome” OR “microbiota” OR “microflora”. All studies published from the inception of the databases until August 2020 were taken into consideration. The reference lists of the identified papers were also searched for further articles.

### 2.2. Eligibility Criteria

Both observational and interventional studies that evaluated the microbiome of the skin, peripheral blood, and/or gastrointestinal tract in rosacea were eligible. Only English-language studies that used high-throughput sequencing methods (culture-independent microbiome studies) were included. Abstracts (no full-text articles available in the database), not original studies (including reviews and meta-analyses), animal studies, case reports, and editorials were excluded from the analysis.

### 2.3. Study Selection and Data Extraction

Two authors (KT and MŻ) independently performed the database search and screening of the eligible papers. In case of doubt, discrepancies were discussed until a consensus was reached. The following data was extracted from each eligible study: author(s) and year of publication; country where the study was carried out; type of microbiome studied (skin/blood/gut); characteristics of rosacea subjects (number, mean age ± standard deviation (SD), gender, type of rosacea); characteristics of control group (number, mean age ± SD and gender of controls); methodology of the study (sample collection, transportation and storage, DNA extraction, microbiome analysis technique, sequencing target, sequencing platform, data analysis platform, and reference sequences database); results of the α- and β-diversity analysis; alterations in the composition of the skin, blood, or gut microbiome in rosacea.

## 3. Results

### 3.1. Search Results

**Figure 1:**
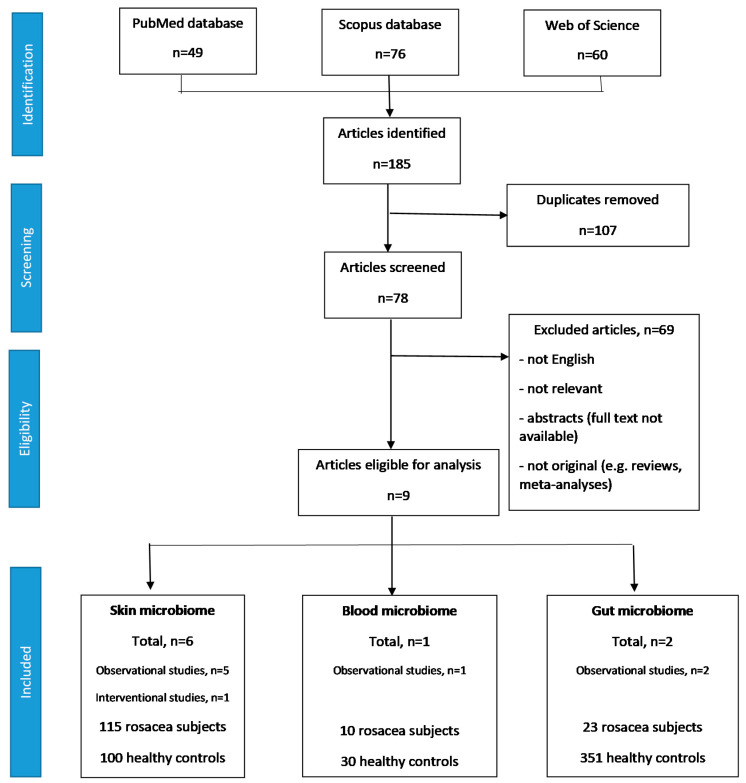
The PRISMA flowchart of literature search and selection.

The literature search retrieved a total number of 185 articles. After the exclusion of duplicates, not relevant, not original, not English-written, and not full text papers, a total number of nine papers were found to be eligible for analysis [12,13,14,15,16,17,18,19,20]. Eight studies were observational [12,13,14,15,16,18,19,20], and one study was interventional [17]. The skin microbiome was investigated in 6 papers, in a total number of 115 rosacea subjects and 100 healthy volunteers. One article reported the blood microbiome alterations in 10 rosacea subjects in comparison to 30 healthy volunteers [18], and two studies focused on the gut microbiota in a total of 23 rosacea patients (compared to 351 healthy controls) [19,20]. The PRISMA flow chart of the literature search and selection is presented in Figure 1. The main characteristics of the papers eligible for analysis and the study participants are summarized in Table 1. The methodology of each study is presented in Table 2. The differences in the microbial α- and β-diversity between rosacea and healthy subjects, are presented in Table 3.

### 3.2. Skin Microbiome in Rosacea

#### 3.2.1. Study Characteristics

Alterations of the skin microbiome in rosacea were the subject of six studies [12,13,14,15,16,17]. Four studies had an observational design, and compared the composition of skin microbiome in rosacea patients to healthy (rosacea-free) individuals [12,13,14,16]. The study by Murillo et al. [12] was focused on Demodex-associated microbiota (Demodex was collected by standardized skin surface biopsies from rosacea and control subjects, and the bacteria from each mite were characterized by a molecular, culture-independent method). The study by Thompson et al. [15] (observational) involved the same rosacea subjects as the study by Rainer et al. [14], but the comparison group included acne patients. One study (Woo et al. [17]) had an interventional design, and compared the skin microbiome changes in rosacea subjects before and after treatment with oral doxycycline. All of the studies assessed the alterations in bacterial microbiome. In addition, the fungal microbiome was investigated in one study (Wang et al. [16]). Characteristics of the participants and study methodology are summarized in Table 1 and Table 2, respectively.

#### 3.2.2. Skin Microbiome α- and β-Diversity in Rosacea

The diversity of the microbiome may be defined in several ways. The α-diversity, referred to as intra-sample diversity, measures the evenness and richness of microbiota in a given environment, while β-diversity, referred to as inter-sample diversity, reflects the existing between-subject differences in microbial composition over time or by location. The skin microbial α- and β-diversity in untreated rosacea subjects was assessed in 4 studies [13,14,15,16]. Wang et al. [16] found increased bacterial α-diversity in PPR compared to healthy controls. No statistically significant differences in the α-diversity between rosacea and healthy subjects were found in the studies by Zaidi et al. [13] (comparison between monozygotic twin pairs with and without rosacea) and Rainer et al. [14] (α-diversity higher in rosacea patients, but not statistically significant). Thompson et al. [15] observed significantly decreased microbial α-diversity in rosacea subjects, in comparison to acne patients. No significant difference was found in the fungal α-diversity between rosacea subjects and controls [16].

The study by Rainer et al. [14] showed no significant difference in the β-diversity between rosacea patients and controls. On the other hand, the difference in the β-diversity between rosacea and acne patients was statistically significant [15]. The study by Zaidi et al. [13] showed no distinct segregation between the rosacea and healthy subjects, while Wang et al. [16] reported an overlap between ETR and PPR, and incomplete separation from healthy controls. There was no significant difference in the fungal β-diversity between rosacea subjects and controls [16].

#### 3.2.3. Composition of the Skin Microbiome in Rosacea

The study by Murillo et al. [12] investigated Demodex-associated microbiota by collecting standardized skin surface biopsies from rosacea subjects and healthy controls. The authors reported a number of alterations in the microbiome of human Demodex mites. At the phylum level, Proteobacteria and Firmicutes were more abundant, and Actinobacteria were less abundant, in PPR, compared to ETR and healthy controls. At the genus level, Bartonella and Haemophilus were limited to ETR, and Escherichia to PPR. At the species level, Staphylococcus hominis, Streptococcus oralis, Streptococcus pneumoniae, and Ochrobactrum grignonense were specific to both subtypes of rosacea. Duganella zoogloeoides was most represented in ETR, while Acinetobacter pitii was most abundant in PPR.

Other studies investigated the alterations of the skin microbiome more extensively by collecting skin swabs or tape strips from the nose and/or cheeks [13,14,16]. At the phylum level, Firmicutes were more abundant, and Actinobacteria were less abundant in rosacea subjects compared to healthy controls [16]. At the genus level, Gordonia [13], Chryseobacterium [13], and Wautersiella [13] were more abundant, and Cutibacterium [16] and Geobacillus [13] were less abundant in rosacea patients, compared to healthy volunteers. Staphylococcus was increased in ETR [16], and Streptococcus was increased in PPR [16]. At the species level, Cutibacterium acnes [14], Azorhizobium doebereinerae [14], Shewanella algae [14], and Providencia stuartii [14] were less abundant both in ETR, and PPR, compared to healthy controls. Porphyromonas endodontalis, Roseomonas mucosa, and Ruminococcus gnavus were decreased in ETR [14]. Actinomyces europaeus, Corynebacterium kroppenstedtii, Prevotella tannerae, Prevotella intermedia, and Campylobacter ureolyticus were increased, while Cutibacterium granulosum, Dysgonomonas gadei, and Anoxybacillus kestanbolensis were decreased in PPR [14]. When compared to acne patients, at the phylum level, Actinobacteria were increased and Proteobacteria were decreased, while at the species level, Cutibacterium acnes and Serratia marcescens were increased in rosacea subjects [15]. Alterations of the skin microbiome composition in rosacea are summarized in Table 4.

#### 3.2.4. Impact of Antibiotic Treatment on the Skin Microbiome Composition

The literature search retrieved only one study (Woo et al. [17]), in which the composition of the skin microbiome was compared before and after treatment with oral doxycycline, at a dose of 100 mg twice daily for 6 weeks. In the aforementioned study, no significant difference in the α-diversity was found before and after therapy with the systemic antibiotic, as well as with patient’s age or rosacea severity. The analysis of the microbial β-diversity showed mild clustering of samples by patient, and minimal clustering by treatment. In untreated rosacea subjects, the predominant bacterial taxa at the genus level were: *Staphylococcus, Cutibacterium, Pseudomonas, Corynebacterium, Acinetobacter,* and *Snodgrasella*. The main bacterial taxa at the species level were: *Staphylococcus epidermidis, Cutibacterium acnes, Pseudomonas koreensis, Acinetobacter haemolyticus,* and *Snodgrassella alvi*. *C. acnes* was significantly more abundant in patients younger than 60 years and in patients with less severe rosacea (Investigator’s Global Assessment, IGA, score of 3 versus 4). On the other hand, *S. alvi* showed a higher abundance in patients with more severe skin involvement (IGA 4 versus IGA 3). Still, no control group of healthy subjects, with rosacea-free skin, was included in the study. After a 6-week therapy with doxycycline, statistically significant change was achieved for one bacterial species, *Weissella confusa*, the abundance of which was significantly enriched in rosacea skin after treatment.

### 3.3. Blood Microbiome in Rosacea

#### 3.3.1. Study Characteristics

The literature search revealed one study that investigated the blood microbiome in rosacea patients [18]. The research was conducted among 10 Korean females with rosacea, and 30 age- and body mass index-matched healthy women. ETR was the predominant subtype, followed by PPR. However, the exact frequencies of each subtype of rosacea were not reported in the paper. Participant characteristics and study methodology are summarized in Table 1 and Table 2.

#### 3.3.2. Blood Microbiome α- and β-Diversity in Rosacea

The results of the analysis of the blood microbiome α-diversity between rosacea and the control group were dependent of the index used for statistical calculations. There was a marginally significant difference when the phylogenetic diversity measurement (Faith’s phylogenetic diversity) was used. On the other hand, the Shannon index, observed OTUs, and evenness failed to reach statistical significance [18].

β-diversity analysis, with weighted and unweighted UniFrac, showed that the blood microbiome from females with rosacea was distinguishable from that of healthy women. In addition, the blood microbiota from the rosacea and control groups partially clustered separately on a principal coordinates plot [18].

#### 3.3.3. Composition of the Blood Microbiome in Rosacea

At the family level, Chromatiaceae and Fusobacteriaceae were significantly elevated in females with rosacea. At the genus level, Rheinheimera, Sphingobium, Tissierellaceae family unknown genus, Paracoccus, Rhodovulum, Marinobacter, Chthoniobacteraceae family unknown genus, Methanobacterium, Armatimonadaceae family unknown genus, Clostridiaceae family unknown genus, Fusobacterium, and Citrobacter were significantly elevated in rosacea subjects [18]. Alterations of the blood microbiome composition in rosacea are summarized in Table 5.

### 3.4. Gut Microbiome in Rosacea

#### 3.4.1. Study Characteristics

The literature search revealed two studies, both of a case-control design, investigating the composition of gut microbiome in rosacea subjects [19,20]. Both studies were conducted in Asia (South Korea and Taiwan), on a total number of 23 rosacea patients and 361 age- and sex-matched healthy volunteers. Females constituted 95.7% and 97.2% of the subjects in the study and the control groups, respectively. The study by Nam et al. [19] was conducted among younger participants (mean age of rosacea patients 42.58 ± 7.98 years) than the study by Chen et al., [20] (mean age of rosacea patients 49.9 ± 11.3 years). Both studies included patients with ETR and PPR. Details regarding study participants and methodology are presented in Table 1 and Table 2, respectively.

#### 3.4.2. Gut Microbiome α- and β-Diversity in Rosacea

Nam et al., [19] did not find significant difference in the gut microbiome α-diversity between rosacea subjects and healthy controls. Chen et al. [20] observed significantly decreased fecal microbial α-diversity in rosacea patients when the Chao 1 index and observed OTUs were applied for statistical analysis, and no significant difference when the Shannon index was utilized. On the other hand, the results of both studies are consistent in terms of inter-sample diversity of the gut microbiome, and point to a statistically significant difference in the β-diversity between rosacea patients and the controls.

#### 3.4.3. Composition of the Gut Microbiome in Rosacea

At the genus level, Lactobacillales order unknown family unknown genus [19], Rhabdochlamydia [20], CF231 [20], Bifidobacterium [20], Sarcina [20], and Ruminococcus [20] were more abundant, while Peptococcaceae family unknown genus [19], Methanobrevibacter [19], Slackia [19], Coprobacillus [19], Citrobacter [19], Desulfovibrio [19], Lactobacillus [20], Hemophilus [20], Roseburia [20], and Clostridium [20] were less abundant in rosacea subjects, when compared to healthy controls. The results are contradictory in terms of the role of Acidaminococcus and Megasphaera, which were found to be more abundant in rosacea subjects in the study by Nam et al. [19], and less abundant in the research by Chen et al. [20]. Alterations of the gut microbiome composition in rosacea are summarized in Table 6.

## 4. Discussion

The role of skin microbiota in chronic cutaneous conditions is increasingly recognized. The skin microbiome in rosacea has been the subject of six studies, to date. However, it should be taken into account that they differed significantly in methodology. The initial study, investigating the skin microbiota in rosacea using metagenomic techniques, was published in 2014 and focused on investigating the bacteria associated with *Demodex* mites, collected by standardized skin surface biopsies from rosacea and healthy subjects. Hence, this research cannot be considered as an analysis of the whole rosacea-associated skin microbiome. Interestingly, the authors did not identify *Bacillus oleronius* among the *Demodex*-associated microbiota. Further, more comprehensive studies have been published in the last three years (2018–2020). Of these, four studies had an observational design, in three the diversity and composition of skin microbiome in rosacea was compared with that of healthy individuals, and in one with that of acne vulgaris patients (it should be noted, that the acne population was younger and more racially diverse). Although each of the papers showed several significant alterations in the skin microbiome of rosacea patients, the results were largely different in each study. Fungal microbiome of the skin has been the subject of one research paper [16], which did not show any significant differences, in terms of its diversity or composition, between rosacea patients and healthy controls.

*C. acnes* was suggested to have a protective role in healthy individuals by breaking down sebum and, thus, preventing the overgrowth of opportunistic microbes [21]. Prior studies showed depletion of *C. acnes* in facial skin biopsies from rosacea patients [22], which was confirmed in the metagenomic analysis by Rainer et al. [14]. In addition, Wang et al. [16] observed a reduced abundance of *Cutibacterium* at the genus level. *C. granulosum*, which classically colonizes healthy skin, and is supposed to prevent the growth of pathogenic microbes, was found to be depleted in PPR, as well [14]. On the other hand, the abundance of *C. acnes* was increased in rosacea when compared with patients with acne vulgaris [15]. The great interest in the skin microbiome is associated with the hope that its modification, e.g., through the use of topical probiotics or transplantation of the microbiome from healthy individuals, will ameliorate the dermatological condition. Transplantation of *Roseomonas mucosa*, a Gram-negative coccobacilli, onto inflamed skin in atopic dermatitis resulted in a significant improvement of the skin symptoms [23]. Interestingly, *R. mucosa* was found to be depleted in ETR patients [14]. Nevertheless, at this stage, it is difficult to reliably interpret the observed increased or depleted abundance of given microorganisms in rosacea.

A complex connection between the digestive tract, brain, and skin, referred to as the gut—brain—skin axis, is widely appreciated by researchers of various fields of science, but the exact interactions have not yet been fully elucidated. This theory is supported by the observation of improvements of skin conditions following the use of oral probiotics or prebiotics [24]. As rosacea has been linked to small intestine bacterial overgrowth and inflammatory bowel disease, one can hypothesize that the gut microbiota may play a role in the pathophysiology of the disease [25]. There is also some evidence in the literature of a distinct gut microbiome composition in patients with rosacea. The intestinal microbiome of rosacea patients has been so far investigated in two studies [19,20]. Although the results are inconsistent in terms of α-diversity, both studies point to significant differences in the gut microbiota β-diversity between rosacea patients and healthy volunteers. Nam et al. [19] found that *Methanobrevibacter, Slackia, Coprobacillus, Citrobacter, Desulfovibrio,* and *Peptococcaceae* family unknown genus were decreased, while *Megasphaera, Acidaminococcus* and *Lactobacillales* order unknown family unknown genus were increased in rosacea patients. On the other hand, Chen et al. [20] found elevated abundance of *Rhabdochlamydia, CF231, Bifidobacterium, Sarcina, and Ruminococcus,* and reduced abundance of *Lactobacillus, Megasphaera, Acidaminococcus, Hemophilus, Roseburia,* and *Clostridium*. As may be noticed, the results of these two studies do not coincide, and are even contradictory for some genera (*Megasphaera* and *Acidaminococcus*). The exact function of the aforementioned genera in the intestinal microbiome, and their potential role in the pathophysiology of rosacea remains undetermined.

A relatively innovative approach is the assessment of the composition of the blood microbiome. Metagenomics offers the possibility of exploring the presence of microbiota in peripheral blood, which may constitute a link between the gut and the skin by stimulating cutaneous inflammatory reactions. This issue might have been neglected so far due to the use of classical culture-dependent diagnostic methods. Alterations of the blood microbiota in rosacea patients have been the subject of one piece of research, so far [18]. Yun et al. [18] found an abundance of two bacterial taxa at the family level, and twelve bacterial taxa at the genus level in blood from Korean females with rosacea. Interestingly, *Fusobacterium*, a Gram-negative bacteria, which is supposed to act under certain conditions as an immunologic trigger in the colon, was found to be abundant in blood in rosacea females. *Fusobacterium* has already been linked to active ulcerative colitis (UC) [26] and colorectal carcinogenesis [27]. On the other hand, prior studies have established a similar genetic background behind UC and rosacea [25]. At the genus level, *Rheinheimera*, a Gram-negative aerobic bacteria belonging to the *Chromatiaceae* family, was found to be the most abundant in blood. Lypopolysaccharide from the outer membrane of Gram-negative bacteria may stimulate innate immunological responses [28]. Nevertheless, the exact role of *Rheinheimera* in rosacea subjects has not been elucidated yet.

Blood microbiota might constitute a link between the gut, and skin inflammation. Notably, the composition of the blood microbiome in rosacea, reported by Yun et al. [18], did not correspond to the alterations of the gut microbiota, reported by Nam et al. [19] and Chen et al. [20]. This may be associated with different study populations, and warrants further investigation. Undoubtedly, there is a need for further research to unravel the mechanisms involved in the gut–skin interaction.

The studies discussed in the current review have several meaningful limitations. First, the number of rosacea subjects in each study is very small, and ranges from 10 to 36 patients. It should be taken into consideration, that there is a huge diversity of microbiomes among individuals. The composition of the microbiome may also be influenced by numerous other factors, which cannot be easily controlled in experimental settings. Therefore, a vast pool of patients is required to define meaningful differences, and the studies conducted to date have not evaluated enough patients to draw reliable conclusions. Moreover, despite the fact that rosacea is more prevalent in white females, the majority of studies were carried out among Asian participants [16,17,18,19,20]. Undoubtedly, genetic factors may impact the composition of the microbiome. Nearly all of the studies included, in varying proportions, patients with ETR and PPR. Various percentages of rosacea subtypes may influence the results of the research. On the other hand, statistical analysis for each subtype is not reliable because of the such low numbers of study participants. In addition, only one of the studies included in the analysis investigated fungal microbiome [16]. Further studies focused on viruses and fungi are needed in order to fully characterize the microbiome in patients with rosacea. Moreover, in the aforementioned studies, the V3–V4 region of the 16S rRNA was the most common sequencing target used for investigating the microbiome of the skin, blood, and intestinal tract. In the study by Meisel et al. [29], skin commensals, including *Cutibacterium*, were found to be poorly captured by sequencing of the V4 region of 16S rRNA. To the best of our knowledge, whole metagenome shotgun sequencing, a much more accurate technique for microbiome characterization, has not yet been utilized in rosacea. Another crucial aspect, that should be considered in research on the role of microorganisms in disease pathogenesis is the “metabolome”. Microbial enzymes and altered metabolism may play a role in disease development, and their evaluation may be even more important for unraveling the pathogenesis than the detection of increased, or decreased, abundance of single bacterial species. Therefore, assessment of the “metabolome” of the microbiome may constitute a pivotal direction for future research.

## 5. Conclusions

The metagenomic approach has revolutionized our perspective on the pathogenesis of chronic skin conditions. The efficacy of antibiotics and ivermectin in the treatment of rosacea constitutes a premise for research on the role of microorganisms in this condition. There is an increasing number of studies suggesting that changes in the skin, blood, and gut microbiome may be associated with rosacea development. Although several studies highlighted significant differences in the microbiota composition between rosacea subjects and healthy controls, their results are inconsistent, or even contradictory in some cases. Most importantly, however, the studies conducted to date do not evaluate enough subjects to draw reliable conclusions, which may be potentially implemented in daily practice. Hence, there is a need for further research to elucidate the diagnostic and therapeutic significance of microbiota alterations in rosacea. Future studies require a vast pool of patients, preferentially of Caucasian origin as this population is predominantly affected by rosacea, and should separately assess the microbiome in ETR and PPR. Research on the “metabolome” should also be a next step in identifying a potential link between the microbiome and rosacea development.

## Figures and Tables

**Table 1:** Studies included in the analysis.

Study/Country	Rosacea					Type of Rosacea			Control Group	Control					Remarks
	Number		Age (Mean ± SD) (Years)		Females (%)	ETR (%)	PPR (%)	Other (%)		Number		Age (Mean ± SD) (Years)		Females (%)	
Skin microbiome															
Murillo et al., 2014 [12] */Germany	30	50.86 ± 11.2 (ETR) 52.82 ± 13.08 (PPR)		N/A		15 (50.0)	15 (50.0)	-	Healthy volunteers	17	52.82 ± 13.08		N/A		Age- and sex-matched controls
Zaidi et al., 2018 [13]/USA	18	37.83 ± 10.62		17 (94.4)		N/A	N/A	N/A	Healthy twins	42	36.36 ± 17.27		37 (88.1)		Twins discordant for rosacea
Rainer et al., 2020 [14] **/USA	19	48.5 ± 12.6		14 (73.7)		11 (57.9)	6 (31.6)	2 (10.5) (ETR/PPR overlap)	Healthy volunteers	19	N/A		N/A		Age-, sex-, and race-matched controls
Thompson et al., 2020 [15] **/USA	19	48.5 ± 12.6		14 (73.7)		11 (57.9)	6 (31.6)	2 (10.5) (ETR/PPR overlap)	Acne subjects	8	N/A		7 (87.5)		
Wang et al., 2020 [16]/China	36	N/A		N/A		21 (58.3)	15 (41.7)	-	Healthy volunteers	22	N/A		N/A		Age- and sex-matched controls
Woo et al., 2020 [17]/South Korea	12	N/A		11 (91.7)		12 (100.0)	-	-	Same group after taking oral antibiotics	12	N/A		11 (91.7)		
Blood microbiome															
Yun et al., 2019 [18]/South Korea	10	N/A		10 (100.0)		N/A	N/A	N/A	Healthy volunteers	30	N/A		30 (100.0)		Age-, sex- and BMI-matched controls
Gut microbiome															
Nam et al., 2018 [19]/South Korea	12	42.58 ± 7.98		12 (100)		6 (50.0)	2 (16.7)	4 (33.3)	Healthy controls	251	43.02 ± 8.23		251 (100)		Age- and sex-matched controls
Chen et al. 2020 [20]/Taiwan	11	49.9 ± 11.3		10 (90.9)		4 (36.3)	7 (63.7)	-	Healthy controls	110	50.6 ± 10.2		100 (90.9)		Age- and sex- matched controls

ETR—erythroteleangiectatic rosacea. PPR—papulopustular rosacea. BMI—body mass index. N/A—not available. * The study by Murillo et al. [12] investigated Demodex-associated microbiota in rosacea subjects compared to healthy controls. ** The same patients with rosacea and controls were included in the studies by Rainer et al. [14] and Thompson et al., [15]. The study by Thompson et al. [16] was extended by an addition of 8 acne subjects matched to 8 controls.

**Table 2:** Methodology of the included studies.

Study	Sample	Sample Transportation and Storage Until Analysis	DNA Extraction	Microbiota Analysis Technique	Sequencing Target	Sequencing Platform	Data Analysis Platform	Reference Sequences Database
Skin microbiome								
Murillo et al., 2014 [12] *	Standardized skin surface biopsies on the malar crease	stored at −80 °C	QIAmp DNA Mini kit	16S rRNA gene sequencing	-	Real-time PCR	ChromasPro	BLASTn nucleotide collection database
Zaidi et al., 2018 [13]	Sebutape strips from bilateral malar cheeks	N/A	MO-BIO PowerSoil DNA Isolation Kit	16S rRNA gene sequencing	V3-V4	Illumina MiSeq	QIIME	Greengenes database
Rainer et al., 2020 [14] **	Skin swabs of the nose and bilateral cheeks	Sample tube containing Amies medium, stored at −80 °C	Zymo fecal DNA kit	Bacterial 16S rRNA gene sequencing	V3-V4	Illumina MiSeq platform	QIIME1/MetaStats 2.0	Greengenes database
Thompson et al., 2020 [15] **	Skin swabs of the nose and bilateral cheeks	Sample tube containing Amies medium, stored at −80 °C	Zymo fecal DNA kit	Bacterial 16S r RNA gene sequencing	V3-V4	Illumina MiSeq platform	QIIME1/MetaStats 2.0	Greengenes database
Wang et al., 2020 [16]	Skin swabs from bilateral cheeks	N/A	Qiagen DNA extraction kit	ITS1 and 16S rRNA gene sequencing	N/A	Illumina HiSeq 2500 platform	QIIME 1.7.0	N/A
Woo et al., 2020 [17]	Skin swabs of the nose and bilateral cheeks	N/A	ZR Fecal DNA MiniPrep	16S rRNA gene sequencing	V3-V4	Illumina HiSeq platform	CD-HIT-OUT analysis program QIIME v1.9	BLASTN v2.4.0 National Center for Biotechnology Information 16S
Blood microbiome								
Yun et al., 2019 [18]	Whole blood collected by peripheral vein puncture	Stored at −4 °C	G-DEX IIb Genomic DNA Extraction Kit for Blood	16S rRNA gene sequencing	V3-V4	Illumina MiSeq platform	QIIME2	GreenGenes database
Gut microbiome								
Nam et al., 2018 [19]	stool	N/A	MO-BIO PowerSoil DNA Isolation Kit	16S rRNA gene sequencing	V3-V4	Illumina MiSeq platform	QIIME 1.9	Greengenes 13_8 database
Chen et al., 2020 [20]	stool	Transferred by using cooler bags, stored at −20 °C	Qiagen DNA isolation kit	Bacterial 16S rRNA gene sequencing	V3-V4	Illumina MiSeq 2000 platform	USEARCH	Greengenes 13_5 database

* Demodex mites were initially collected by standardized skin surface biopsies from rosacea and control subjects and then the microbiota from each mite were characterized by 16S rRNA sequencing. ** The same patients with rosacea and controls were included in the studies by Rainer et al. [14] and Thompson et al., [15]. The study by Thompson et al. [15] was extended by an addition of 8 acne subjects matched to 8 controls.

**Table 3:** α- and β-diversity of microbiota in rosacea.

Study	α-Diversity	β-Diversity
Skin microbiome		
Zaidi et al., 2018 [13]	-No significant difference between monozygotic twin pairs with and without rosacea -Negative association with the severity of rosacea	-No distinct segregation between rosacea subjects and healthy controls -Greater weighted UniFrac distance between siblings in which one has rosacea than between siblings with rosacea and siblings without rosacea (not statistically significant) -monozygotic twins have more similar facial microbiome than dizygotic twins
Rainer et al., 2020 [14]	-Mean microbial α-diversity (total and within individual rosacea subtypes) higher in rosacea subjects than in controls, but the difference was not significant	-No significant difference (total and with regards to individual rosacea subtypes)
Thompson et al., 2020 [15]	-Significantly decreased skin microbial diversity in rosacea subjects, compared to acne patients	-Significant difference between rosacea patients and acne patients
Wang et al., 2020 [16]	-Bacterial microbiome: increased bacterial diversity in PPR, compared with controls -Fungal microbiome: no significant difference between rosacea subjects and healthy controls	-Bacterial microbiome: overlap between ETR and PPR, and incomplete separation from healthy controls -Fungal microbiome: no significant differences between rosacea subjects and controls
Woo et al., 2020 [17]	-no significant difference before and after treatment -no significant difference with age (≤60 versus >60) and rosacea severity (IGA3 versus IGA4)	-mild clustering of samples by patient and minimal clustering of samples by treatment
Blood microbiome		
Yun et al., 2019 [18]	-no significant difference (Shannon Index, observed OTUs) -marginally significant difference (Faith’s phylogenetic diversity)	-significant difference (weighted and unweighted UniFrac) -partially separate clustering of the blood microbiota from rosacea subjects and controls (weighted UniFrac)
Gut microbiome		
Nam et al., 2018 [19]	-No significant difference	-Significant difference
Chen et al., 2020 [20]	-Significantly decreased fecal microbial richness (number of observed OTUs and Chao 1) -No significant difference (Shannon Index)	-Significant difference

IGA—Investigator’s Global Assessment. OTUs—operational taxonomic units.

**Table 4:** Skin microbiome alterations in rosacea.

Phylum	Class	Order	Family	Genus	Species
Actinobacteria ↑ [15] ^a^ ↓ [12] ** [16] */**	Actinobacteria	Corynebacteriales	Corynebacteriaceae	Corynebacterium	*Corynebacterium kroppenstedtii* ↑ [14] **
		Actinomycetales	Propionibacteriaceae	Cutibacterium ↓ [16] */**	*Cutibacterium acnes* ↓ [14] ↑ [15] ^a^
					*Cutibacterium granulosum* ↓ [14] **
			Gordoniaceae	Gordonia ↑ [13]	
			Actinomycetaceae	Actinomyces	*Actinomyces europaeus* ↑ [14] **
Bacteroidetes	Bacteroidetes	Bacteroidales	Prevotellaceae	Prevotella	*Prevotella tannerae* ↑ [14] **
					*Prevotella intermedia* ↑ [14] **
			Porphyromonadaceae	Dysgonomonas	*Dysgonomonas gadei* ↓ [14] **
				Porphyromonas	*Porphyromonas endodontalis* ↓ [14] *
	Flavobacteriia	Flavobacteriales	Flavobacteriaceae	Chryseobacterium ↑ [13]	
				Wautersiella ↑ [13]	
Proteobacteria ↓ [15] ^a^ ↑ [12] **	Alphaproteobacteria	Rhizobiales	Xanthobacteraceae	Azorhizobium	*Azorhizobium doebereinerae* ↓ [14] */**
			Brucellaeceae	Ochrobactrum	*Ochrobactrum grignonense* ↑ [12] */**
			Bartonellaceae	Bartonella ↑ [12] *	
		Rhodospirillales	Acetobacteraceae	Roseomonas	*Roseomonas mucosa* ↓ [14] *
	Betaproteobacteria	Burkholderiales	Oxalobacteraceae	Duganella	*Duganella zoogloeoides* ↑ [12] *
	Gammaproteobacteria	Alteromonadales	Shewanellaceae	Shewanella	*Shewanella algae* ↓ [14] */**
		Enterobacterales	Morganellaceae	Providencia	*Providencia stuartii* ↓ [14] */**
			Yersiniaceae	Serratia	*Serratia marcescens* ↑ [15] ^a^
			Enterobacteriaceae	Escherichia ↑ [12] **	
		Pseudomonadales	Moraxellaceae	Acinetobacter	*Acinetobacter pittii* ↑ [12] **
		Pasteurellales	Pasteurellaceae	Haemophilus ↑ [12] *	
	Epsilonproteobacteria	Campylobacterales	Campylobacteraceae	Campylobacter	*Campylobacter ureolyticus* ↑ [14] **
Firmicutes ↑ [12] ** [16] */**	Bacilli	Bacillales	Bacillaceae	Anoxybacillus	*Anoxybacillus kestanbolensis* ↓ [14] **
				Geobacillus ↓ [13]	
			Staphylococcaceae	Staphylococcus ↑ [16] */**(NS)	*Staphylococcus hominis* ↑ [12]*/**
		Lactobacillales	Streptococcaceae	Streptococcus ↑ [16] **	*Streptococcus oralis* ↑ [12] */**
					*Streptococcus pneumoniae* ↑ [12] */**
	Clostridia	Clostridiales	Ruminococcaceae	Ruminococcus	*Ruminococcus gnavus* ↓ [14] *
			Lachnospiraceae	Blautia ↑ [13]	

^a^ acne subjects constituted the comparison group. * erythematoteleangiectatic rosacea (ETR). ** papulopustular rosacea (PPR).

**Table 5:** Blood microbiome alterations in rosacea.

Phylum	Class	Order	Family	Genus
Proteobacteria	Alphaproteobacteria	Sphingomonadales	Sphingomonadaceae	Sphingobium ↑ [18]
		Rhodobacterales	Rhodobacteraceae	Paracoccus ↑ [18]
				Rhodovulum ↑ [18]
	Gammaproteobacteria	Chromatiales	Chromatiaceae ↑ [18]	Rheinheimera ↑ [18]
		Alteromonadales	Alteromonadaceae	Marinobacter ↑ [18]
		Enterobacterales	Enterobacteriaceae	Citrobacter ↑ [18]
Fusobacteria	Fusobacteriia	Fusobacteriales	Fusobacteriaceae ↑ [18]	Fusobacterium ↑ [18]
Firmicutes	Clostridia	Clostridiales	Tissierellaceae	Tissierellaceae family unknown genus ↑ [18]
			Clostridiaceae	Clostridiaceae family unknown genus ↑ [18]
Verrucomicrobia	Spartobacteria	Cthnoniobacterales	Chtoniobacteraceae	Chtoniobacteraceae family unknown genus ↑ [18]
Euryarchaeota	Methanobacteria	Methanobacteriales	Methanobacteriaceae	Methanobacterium ↑ [18]
Armatimonadetes	Armatimonadia	Armatimonadales	Armatimonadaceae	Armatimonadaceae family unknown genus ↓ [18]

**Table 6:** Gut microbiome alterations in rosacea.

Phylum	Class	Order	Family	Genus
Bacteroidetes	Bacteroidia	Bacteroidales	Bacteroidaceae	Bacteroides ↑ [20]
			Prevotellaceae	Prevotella ↓ [20]
				CF231 ↑ [20]
Fusobacteria	Fusobacteriia	Fusobacteriales	Fusobacteriaceae	Fusobacterium ↑ [20]
Proteobacteria	Betaproteobacteria	Burkholderiales	Sutterellaceae	Sutterella ↓ [20]
	Gammaproteobacteria	Pasteurellales	Pasteurellaceae	Haemophilus ↓ [20]
		Enterobacterales	Enterobacteriaceae	Citrobacter ↓ [20] [19]
	Deltaproteobacteria	Desulfovibrionales	Desulfovibrionaceae	Desulfovibrio ↓ [19]
Chlamydiae	Chlamydiae	Chlamydiales	Rhabdochlamydiaceae	Rhabdochlamydia ↑ [20]
Actinobacteria	Actinobacteria	Bifidobacteriales	Bifidobacteriaceae	Bifidobacterium ↑ [20]
		Coriobacteriales	Coriobacteriaceae	Slackia ↓ [19]
Firmicutes	Clostridia	Clostridiales	Clostridiaceae	Sarcina ↑ [20]
				Clostridium ↓ [20]
			Ruminococcaceae	Ruminococcus ↑ [20]
			Lachnospiraceae	Roseburia ↓ [20]
			Peptococcaceae	Peptococcaceae family unknown genus ↓ [19]
	Bacilli	Lactobacillales	Lactobacillaceae	Lactobacillus ↓ [20]
				Lactobacillales order unknown family unknown genus ↑ [19]
	Negativicutes	Selenomonadales	Veillonellaceae	Megasphaera ↓ [20] ↑ [19]
			Acidaminococcaceae	Acidaminococcus ↓ [20] ↑ [19]
	Erysipelotrichia	Erysipelotrichales	Erysipelotrichaceae	Coprobacillus ↓ [20]
Euryarchaeota	Methanobacteria	Methanobacteriales	Methanobacteriaceae	Methanobrevibacter ↓ [19]
